# Pathogenicity Analysis of Weaned Piglets Challenged With Novel Emerging Senecavirus A in Fujian, China

**DOI:** 10.3389/fvets.2021.694110

**Published:** 2021-07-07

**Authors:** Cun Liu, Yanhan Liu, Xiubo Li, Lin Liang, Shangjin Cui

**Affiliations:** ^1^Institute of Animal Sciences, Chinese Academy of Agricultural Sciences, Beijing, China; ^2^Shandong Provincial Center for Animal Disease Control, Ji'nan, China; ^3^Beijing Observation Station for Veterinary Drug and Veterinary Biotechnology, Ministry of Agriculture, Beijing, China

**Keywords:** emerging disease, pathogenicity, vesicular disease, Senecavirus A, weaned piglets

## Abstract

In order to evaluate the pathogenicity of Senecavirus A (SVA) to weaned piglets preliminarily, 28-day-old weaned piglets were challenged with SVA by intramuscular injection. The clinical manifestations, antibody levels, and tissue viral load of infected piglets were detected. The results indicated that the piglets challenged with SVA CH/FuJ/2017 showed drowsiness, lameness, oral blisters, diarrhea, and other clinical signs. Lesions on the hooves were observed. Red spots or plaques were initially observed on the hoof and then developed into blisters that cracked and gradually formed scab. The symptoms and signs were relieved after 8 days post-infection (dpi). The sentinel piglet, feeding together with the challenged piglets, showed similar clinical signs with the challenged piglets after 3 dpi. Monitoring of antibody levels showed that anti-SVA antibody could be detected at 5 dpi by competition enzyme-linked immunosorbent assay (cELISA) method, and neutralizing antibody could be detected after 7 dpi. Analysis of viral tissue distribution and viral load indicated that SVA could replicate in the liver, spleen, lung, kidney, and lymph node. In all, Senecavirus disease was successfully replicated by SVA CH/FuJ/2017 isolate, which verified the clinical manifestations of SVA infection in weaned piglets, and provided a foundation for further SVA pathogenesis and vaccine development.

## Introduction

Senecavirus A (SVA), also known as Seneca Valley virus (SVV), is a single-stranded positive sense RNA virus belonging to *Senecavirus* genus, *Picornaviridae* family (1). The mature virion of SVA is a non-enveloped icosahedral particle with a diameter of 25~30 nm. The SVA genome is ~7.2 kb in length and contains a unique open reading frame (ORF) that is flanked by 5′ and 3′ untranslated regions (UTRs), with the 3′-UTR followed by a poly (A) tail. The single ORF present in the SVA genome encodes a large polyprotein that is cleaved by virus-encoded proteases into 12 mature viral proteins (5′-L–VP4–VP2–VP3–VP1–2A−2B−2C−3A−3B−3C−3D-3′) (2, 3).

SVA was originally isolated from PER.C6 as a cell culture contaminant in America in 2002 (3). The pieces of evidence that SVA has been associated with porcine idiopathic vesicular disease (PIVD) were provided by these sporadic cases that occurred in USA and Canada (4, 5). Since 2015, an increasing number of cases of vesicular diseases in pigs, which were later proven to be caused by SVA infection, were reported in many countries including USA, Brazil, China, Colombia, and Thailand (1, 5–10). In China, SVA infection was firstly reported in 2015 (1). Since then, the outbreak of SVA infection was detected in several provinces, including Guangdong, Henan, Heilongjiang, Hubei, and so on (11). Phylogenetic analysis of SVA isolates in China showed that the isolates in China could be divided into five groups, which were closely related to the isolates from the United States and Canada (12).

In our previous study, an emerging SVA, named CH/FuJ/2017 (GenBank No. MH490944), was isolated from vesicular fluid from a swine herd in which pigs were compulsorily vaccinated with foot-and-mouth disease virus (FMDV) vaccine. Phylogenetic analysis showed that SVA CH/FuJ/2017 strain was closely related to the American SVA isolates (13). In the present study, the pathogenicity of SVA CH/FuJ/2017 in weaned pigs was evaluated to understand the characteristics of vesicular disease caused by SVA infection.

## Methods

### Cell Cultivation and Virus Proliferation

Baby hamster Syrian kidney 21 cells (BHK-21) were cultured at 37°C with 5% CO_2_ in Dulbecco's modified Eagle's medium (DMEM; Fisher Scientific, Loughborough, UK) supplemented with 8% horse serum. BHK-21 was infected with SVA CH/FuJ/2017 according to the proportion of one thousandth of the volume of cell culture medium. The virus was collected when cytopathic effect (CPE) was more than 70%.

### Animal Experiment Design

In this study, 28-day-old weaned piglets were provided by Zhejiang Mebolo Swine Breeding Co., Ltd. Prior to the experimental infections, serum samples were collected from the unchallenged pigs for serologic test using Seneca Valley virus A (SVA) Antibody Test Kit (Biovet Inc., Canada).

The virus with 10^6^ TCID_50_/ml titer was used for experimental infection. To ensure the success of the infection, weaned pigs (*n* = 5) in the challenged group were infected with SVA CH/FuJ/2017 strain *via* inoculation intranasally (2 ml) and intramuscularly (3 ml). One sentinel pig was co-housed with the infected pigs. The piglets for normal control (*n* = 5) were isolated during feeding. All weaned pigs within the two groups were kept in individual rooms that were designed as the mechanically ventilated negative-pressure animal house. Relative humidity and temperature were maintained at 65–80% and 24°C.

Clinical signs and the rectal temperature of pigs inoculated with SVA CH/FuJ/2017 were monitored daily for 14 days. Serum samples were collected at 1, 3, 5, 7, 10, and 14 days post-infection (dpi) for the evaluation of humoral response. In order to observe the pathological damage caused by virus infection in the acute infection period, two pigs were euthanized under pentobarbital sodium anesthetic at 7 dpi. The rest of the pigs were euthanized with the same procedure at 14 dpi. Tissue samples, including heart, liver, spleen, lung, kidney, brain, and lymph nodes, were collected for the distribution of viral load and histopathological examination.

### Detection of Senecavirus a Genomic RNA

Total RNA of tissues and serum samples was extracted using TRIzol reagent according to the manufacturer's instructions. cDNA was synthesized with TransScript One-Step gDNA Removal and cDNA Synthesis SuperMix (Beijing QuanShiJin Biotechnology Co., Ltd.). The real-time RT-PCR (RT-qPCR) for SVA detection was used for the detection of SVA genomic RNA according to previous research (14).

### Histopathological Examination

Tissues for histopathological examination were fixed in 4% paraformaldehyde; all tissues were embedded in paraffin. Then, pathological tissue sections were stained with hematoxylin and eosin. Finally, all pathological tissue sections were observed under a microscope.

### Detection of Serum Antibody

Virus neutralization test and competition enzyme-linked immunosorbent assay (cELISA) were performed for the analysis of humoral response of SVA-infected pigs. cELISA was conducted following the manufacturer's instruction of Seneca Valley virus A (SVA) Antibody Test Kit (Biovet Inc., Canada).

### Statistical Analysis

The statistical analysis was carried out by GraphPad software with *t*-test or one-way ANOVA. Statistical significance was indicated *p*-value < 0.05.

## Results

### Clinical Presentation of the Piglets Inoculated With Senecavirus A

**Figure 1:**
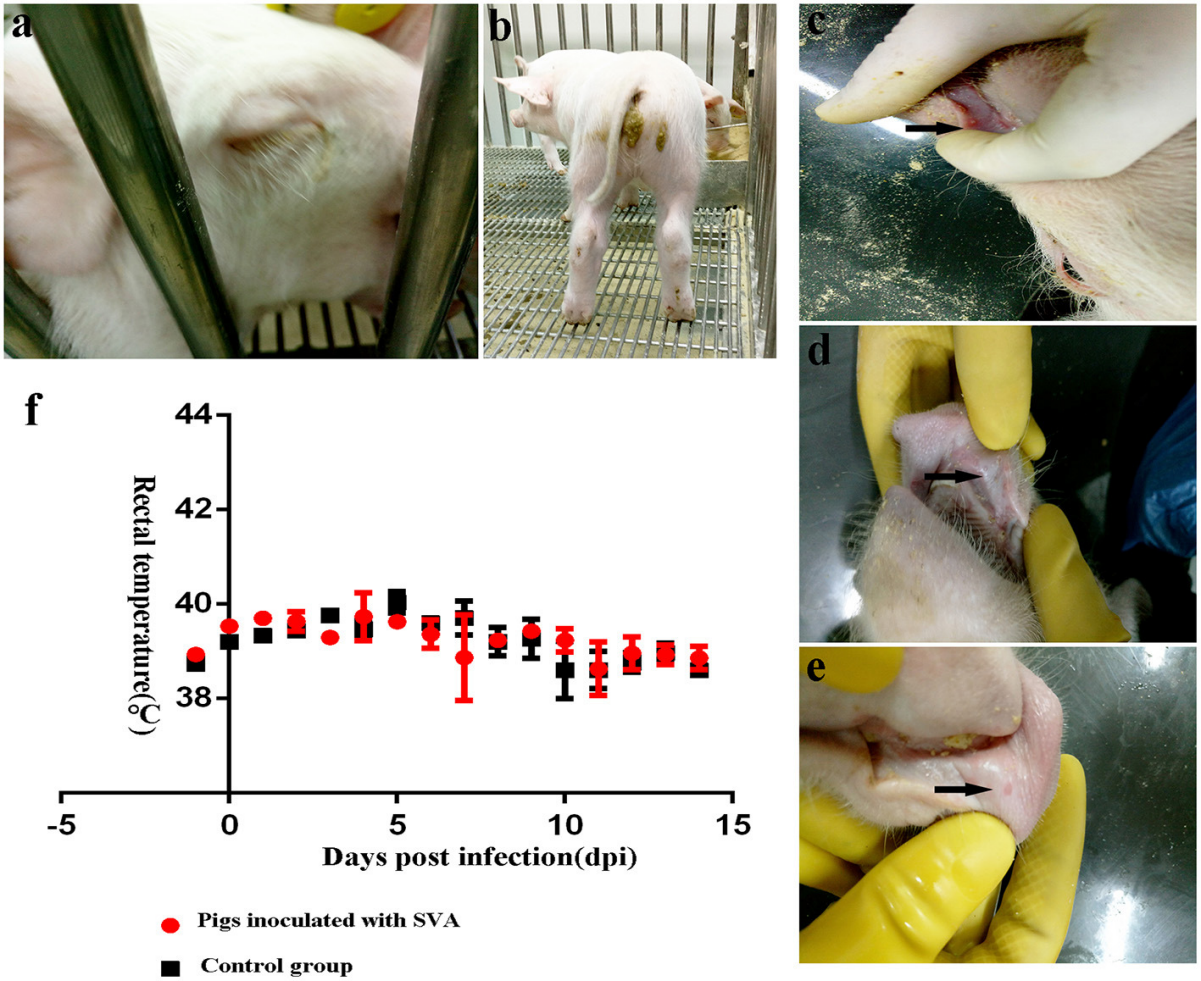
Clinical signs of weaned pigs challenged with Senecavirus A (SVA) CH/FuJ/2017. **(a)** Lethargy was observed at 2 days post-infection (dpi). **(b)** Diarrhea caused by SVA infection was found at 3 dpi. **(c–e)** The development of SVA-induced vesicle on the oral mucosa (black arrow). Small fluid-filled vesicles were firstly observed at 3 dpi and rapidly ruptured and recovered. **(f)** The rectal temperature was monitored daily. Persistent high fever was not observed, and there was no significant difference in rectal temperature between challenged and unchallenged pigs.

All piglets (*n* = 5) in the challenged group had Senecavirus disease after SVA challenge. Lethargy and lameness of piglets were observed at 2 dpi (Figure 1a). Diarrhea was first observed at 3 dpi and began to recover at 8 dpi (Figure 1b). The piglets unexceptionally presented small fluid-filled vesicles in the oral mucosa at 3 dpi and ruptured ulcer, which recovered rapidly, was also observed (Figures 1c–e). Persistently high fever was not observed in SVA-infected piglets (Figure 1f). Occasionally, the rectal temperature of a few piglets was over 40°C (Supplementary Table 1). No pig died after SVA infection throughout the experiment.

### Hoof Lesions Caused by Senecavirus a Infection

**Figure 2:**
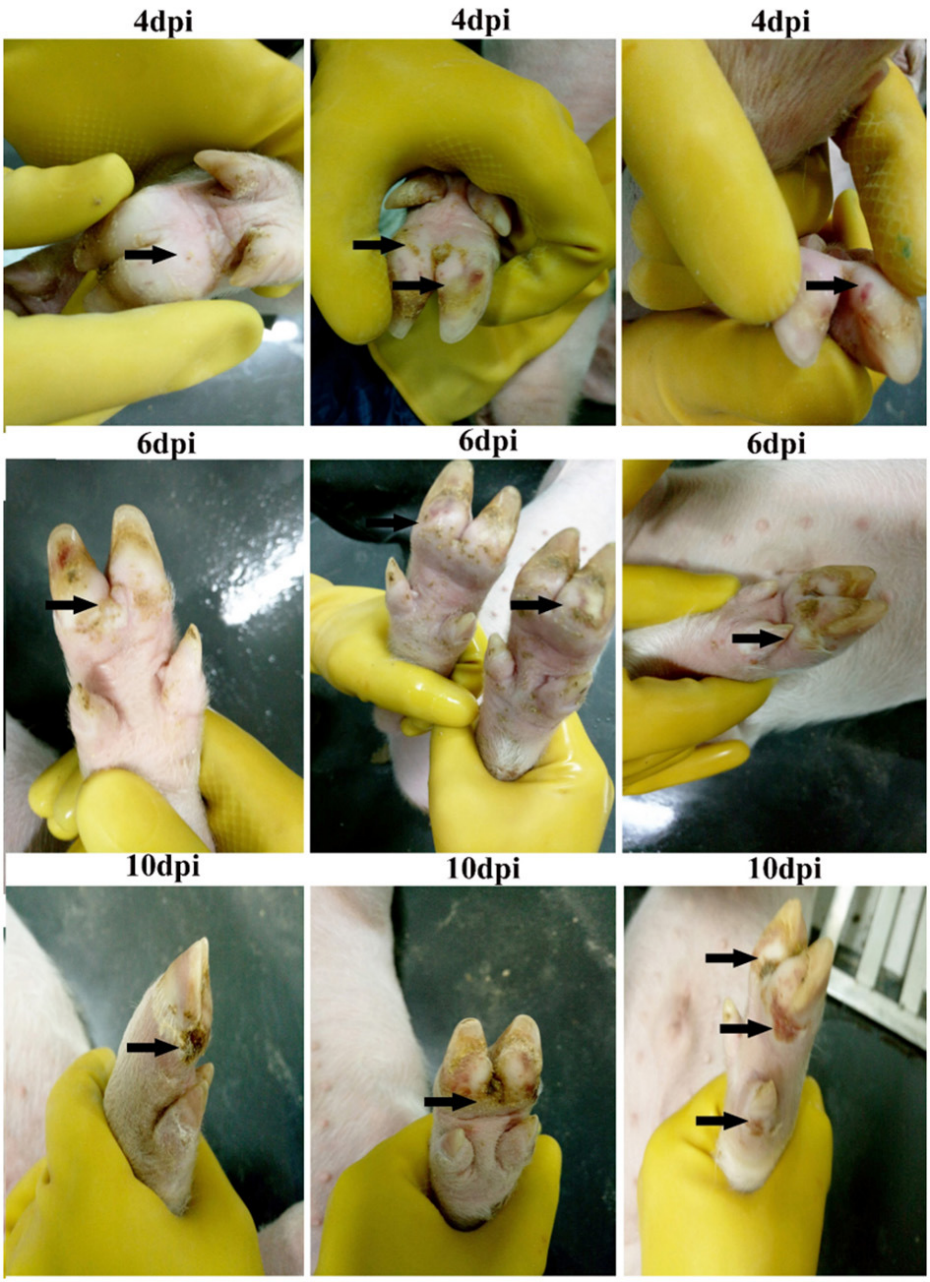
Progression of Senecavirus A (SVA)-induced vesicular disease on hooves. Red spots were firstly observed on hooves [4 days post-infection (dpi)] and then rapidly developed into fluid-filled vesicles (6 dpi). Finally, the vesicles ruptured and left ulcerated lesions on the hooves.

SVA infection induced hoof lesions in piglets. The lesions mainly showed in toes, interphalangeal spaces, coronary band, and dew claw. There are three stages in the development of hoof lesions. Initially, red spots or patches were observed at 4 dpi. Secondly, the red spots or patches rapidly developed into fluid-filled vesicles at 6 dpi (Figure 2). Finally, the fluid-filled vesicles broke up and gradually developed into scab lesions (at 10 dpi), which were left on hooves, persisting until the end of the experiment (Figure 2).

### Signs of Sentinel Piglets

To investigate the transmission characteristic, sentinel pigs were used to verify the transmission route. During the test, we found that co-housed sentinel pigs and sick pigs shared the water dispenser and material trough, and there were such phenomena as touching each other with snout, eating excrement, and biting railings. These provided convenience for the spread of the virus. Sentinel pigs showed similar clinical signs with SVA-infected pigs. Fluid-filled vesicles in the oral mucosa were observed by 3 dpi, and lethargy, lameness, and fluid-filled vesicles on hooves were observed at 4 dpi. Vesicles mainly appeared in the interdigital space and the coronary band. In addition, indirect contact sentinel pigs never showed SVA infection-related clinical signs. These reconfirmed the fact that contact transmission for SVA was the important approach to promote virus circulation in the herd.

### Histopathological Changes

**Figure 3:**
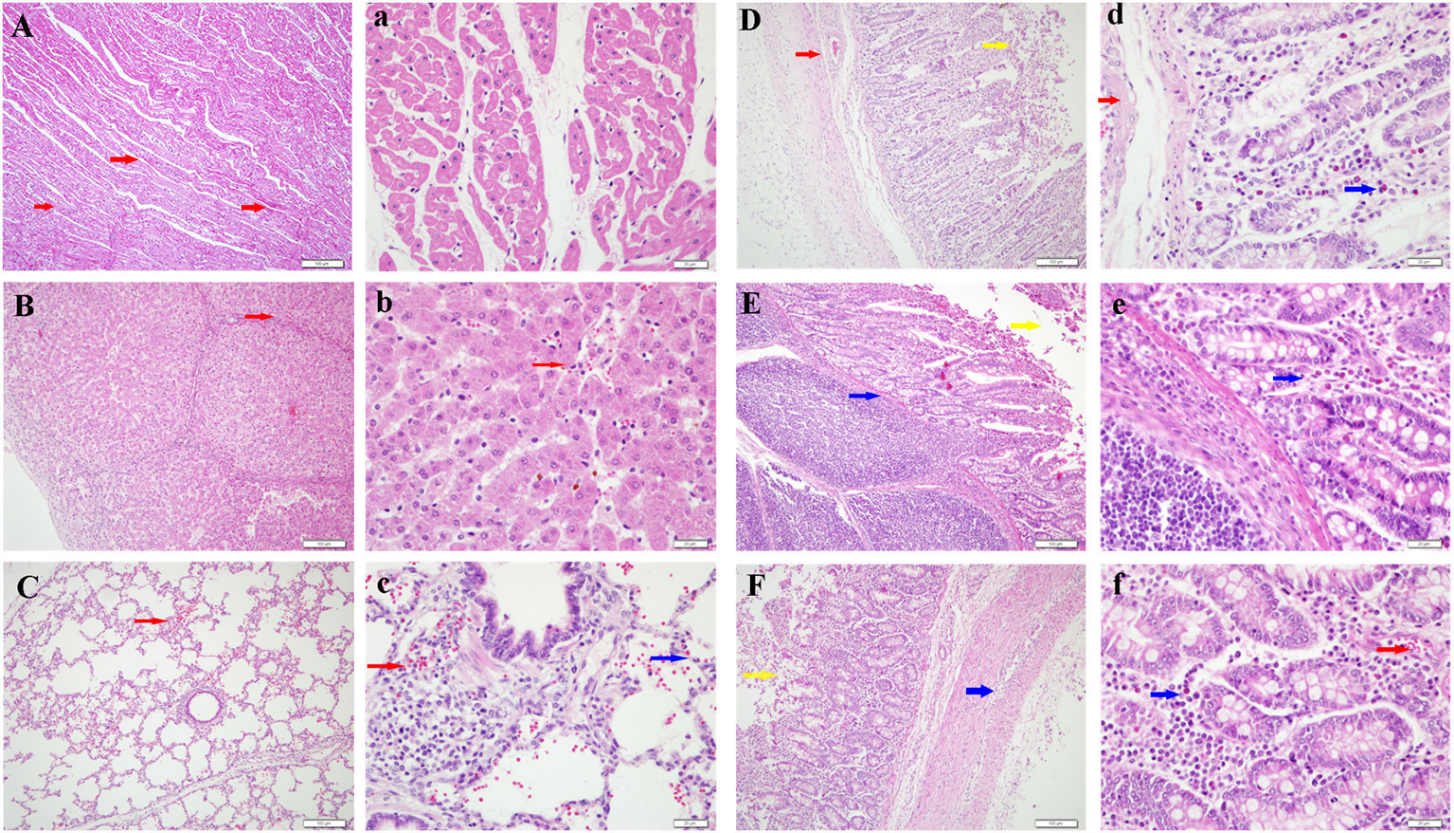
Histological changes in different tissues induced by Senecavirus A (SVA) infection. (**A,a**) Enlargement of the myocardial fiber space, cellular vacuolation, and hemorrhage in the heart (red arrow). (**B,b**) Eosinophilic lesions, cytoplasmic concentration, and scattered distribution of red blood cells (red arrow) in the liver. **(C,c)** Emphysema, congestion (red arrow), and inflammatory cell infiltration (blue arrow) in the lung. Abscission of intestinal epithelial cells (yellow arrow), inflammatory cell infiltration (blue arrow) in the lamina propria, and minor bleeding (red arrow) were observed in the jejunum **(D,d)**, ileum **(E,e)**, and duodenum (**F,f**). Bar, **(A–F)**: 100 μm; **(a–f)**: 20 μm.

SVA-infected piglets showed pathological lesions, such as hemorrhage, edema, and inflammatory cell infiltration, in various organs. Histopathological lesions were predominantly observed in the lung, liver, heart, and small intestine. The virus caused hemorrhage and edema in the heart, emphysema and inflammatory cell infiltration in the lung, eosinophilic lesions in the liver, inflammatory cell infiltration in the kidney, lymphoid hyperplasia in the spleen, and hemorrhage in the lymph nodes. Pathological lesions caused by SVA infection in the small intestine were abscission of intestinal epithelial cells, inflammatory cell infiltration in the lamina propria, and minor bleeding (Figure 3).

### The Viral Distribution Analysis

TaqMan real-time PCR was used for the viral distribution analysis. SVA genomic RNA was detected in the liver, spleen, lung, kidney, and lymph nodes, showing that the virus was able to replicate in various visceral tissues (Supplementary Figure 1). The virus was successfully reisolated with BHK-21 cells (Supplementary Figure 2). Serum samples were collected at 1, 3, 5, 7, 10, and 14 dpi for the assessment of viremia. The SVA genomic RNA was detected in serum samples collected at 1 and 3 dpi. However, no SVA genomic RNA could be detected in serum samples after 5 dpi in our study (Supplementary Table 2).

### Changes of Serum Antibody Levels

**Figure 4:**
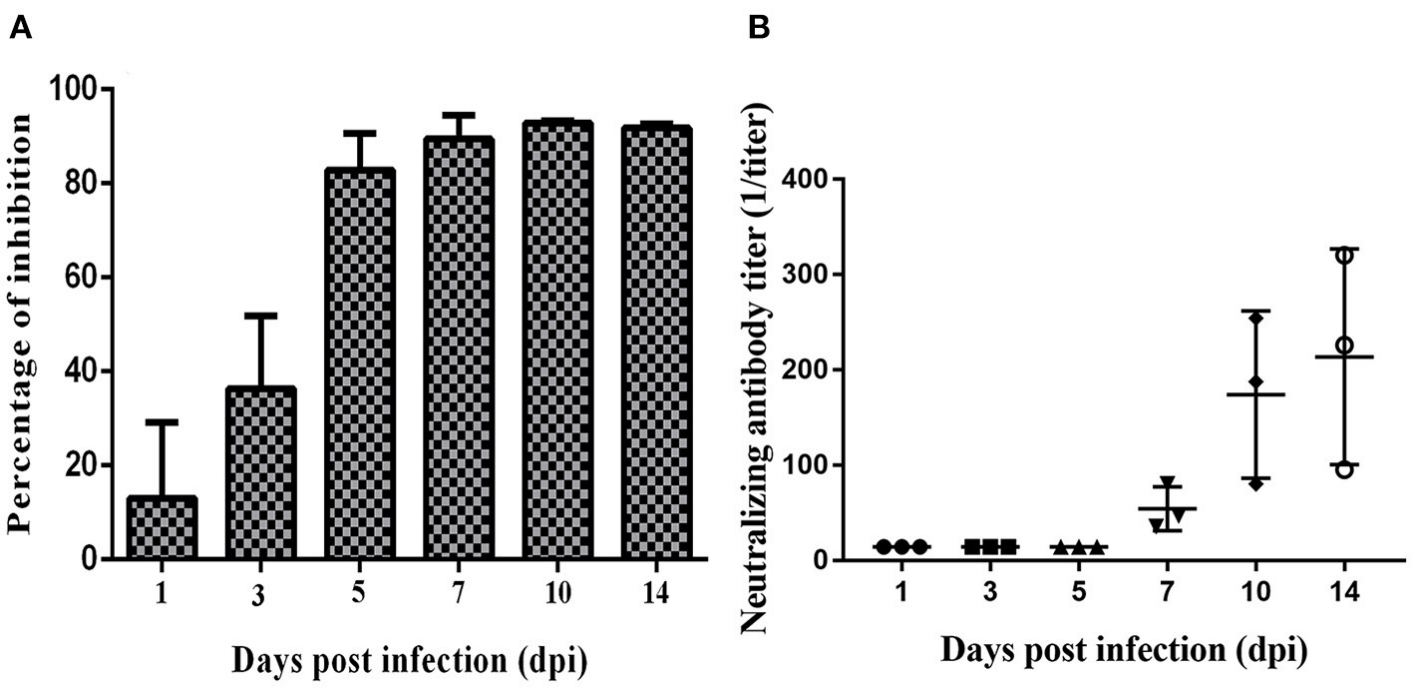
Changes of anti-Senecavirus A (SVA) serum antibody levels during SVA infection. **(A)** Anti-SVA serum antibody detected with cELISA. **(B)** The dynamic change of neutralizing antibody detected with neutralization assay.

The anti-SVA antibody and neutralizing antibody were detected with cELISA and virus neutralization test. Seroconversion was detected at 5 dpi, and it persisted until the end of the experiment (Figure 4A, Supplementary Table 3). However, neutralizing antibody was firstly detected at 7 dpi with a progressively increasing tendency (Figure 4B, Supplementary Table 4). These results suggested that pigs may develop a rapid and robust humoral immune response due to acute SVA infection.

## Discussion

Senecavirus disease is an emerging viral infectious disease in recent years. It is characterized by vesicles on the snout and hooves of pig, which is difficult to distinguish from FMD, swine vesicular disease, and vesicular stomatitis (15, 16). In recent years, more and more cases of Senecavirus disease have been reported around the world. At present, the epidemic situation of Senecavirus disease in China is far from optimistic. According to existing reports, more than half of provinces in China had found SVA infection (17). In addition, 278 out of 2,547 serum samples were detected as SVA antibody positive in Hainan Province (18).

The early isolated SVA strain was non-pathogenic to pig, and experimental infection failed to replicate Senecavirus disease. However, sporadic cases occurring in Canada in 2008 and the United States in 2012 revealed that SVA infection was associated with vesicular disease in pigs (4, 5). Subsequently, successfully replicated Senecavirus disease using contemporary SVA strains further confirmed that SVA was the agent of Senecavirus disease (19, 20). In this study, experimental infection test was performed to evaluate the pathogenicity of SVA CH/FuJ/2017 in weaned piglets. SVA-infected piglets showed lethargy, lameness, diarrhea, and other clinical symptoms until 8 dpi. The lesions on hooves, initially found on 3 dpi, developed from red spots or patches to vesicle and finally formed scab. These clinical presentation was similar to that of fattening pigs infected with SVA in the study by Joshi et al. (19). Diarrhea was an important factor causing death of piglets. In this study, SVA infection caused diarrhea of weaned piglets, which was an important potential threat to suckling piglets. In the experimental infection, it was found that SVA infection did not cause persistent fever in weaned piglets (19). In our study, there was also no significant difference in rectal temperature between SVA-infected pigs and control pigs in the experimental infection. SVA and FMDV belong to the same family of *Picornaviridae*, but FMDV infection could cause a temperature rise in pigs, ranging from 40 to 41°C. Does this difference suggest that there was a difference in the pathogenic mechanism?

In this study, the tissue distribution of SVA was analyzed. SVA genomic RNA was found in the liver, spleen, lung, kidney, and lymph nodes. Other studies have found that SVA genomic RNA could be detected in central nervous system tissues (20). Previous studies found that time to the onset of viremia was short (19, 20). In sows, viremia could be detected within 1 week after the onset of disease, while in suckling piglets, viremia decreased rapidly 1 week after the onset of disease (21). In our study, we found that SVA genomic RNA could not be detected in the serum after 5 dpi. In our study, SVA antibody could be detected in the early stage of SVA infection (5 dpi) with cELISA, and neutralizing antibody was detected after 7 dpi. Previous study showed that neutralizing antibody could be detected as early as 5 dpi (19); however, this detectable difference may be related to our dilution operation. With the increase of antibody titer, the symptoms of infected piglets gradually improved, which also showed that with the activation of the host immune system, SVA was gradually eliminated by the host (22–24). Therefore, it was reasonable to speculate that the early rapid antibody response may be part of the reason for the rapid remission of viremia and the restriction of virus transmission *in vivo*.

## Conclusion

Senecavirus disease was successfully reproduced through experimental infection, and the changes of viremia, viral distribution, and serum antibody changes preliminarily studied in piglets. The results also showed that direct contact was an important way of SVA transmission.

## Data Availability Statement

The original contributions presented in the study are included in the article/Supplementary Material, further inquiries can be directed to the corresponding authors.

## Ethics Statement

The animal study was reviewed and approved by The Science Research Department of the Institute of Animal Science, Chinese Academy of Agricultural Sciences (IAS-CAAS) (Beijing, China) (No. IASCAAS-AE-03, 12-12-2016).

## Author Contributions

SC: conceptualization, funding acquisition, investigation, project administration, supervision, and validation. CL, XL, and LL: data curation and formal analysis. SC and CL: methodology. CL and YL: writing—original draft. SC, CL, and LL: writing—review and editing. All authors reviewed the final draft and agreed with its content and conclusions.

## Conflict of Interest

The authors declare that the research was conducted in the absence of any commercial or financial relationships that could be construed as a potential conflict of interest.
