# Supplementary material for: Pathogenicity Analysis of Weaned Piglets Challenged With Novel Emerging Senecavirus A in Fujian, China
Source: Front Vet Sci. 2021 Jul 7;8:694110. doi: 10.3389/fvets.2021.694110 (PMC8292739; doi:10.3389/fvets.2021.694110)
Supplement: Supplementary file 1 [file Data_Sheet_1.DOCX]

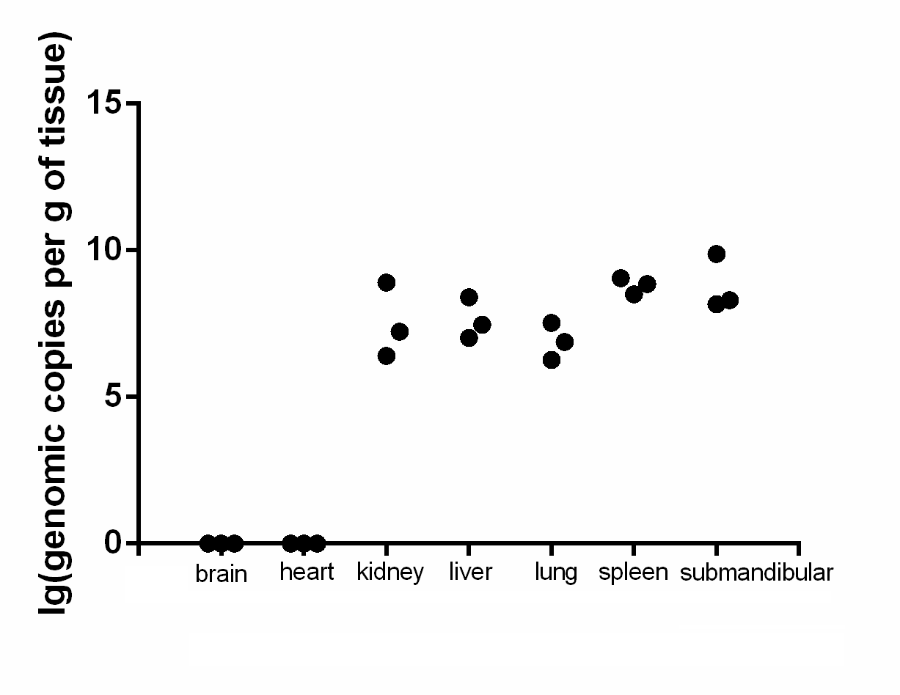


Fig S1 The quantification of SVA genomic RNA in different tissues from SVA infected weaned pigs by RT-qPCR.


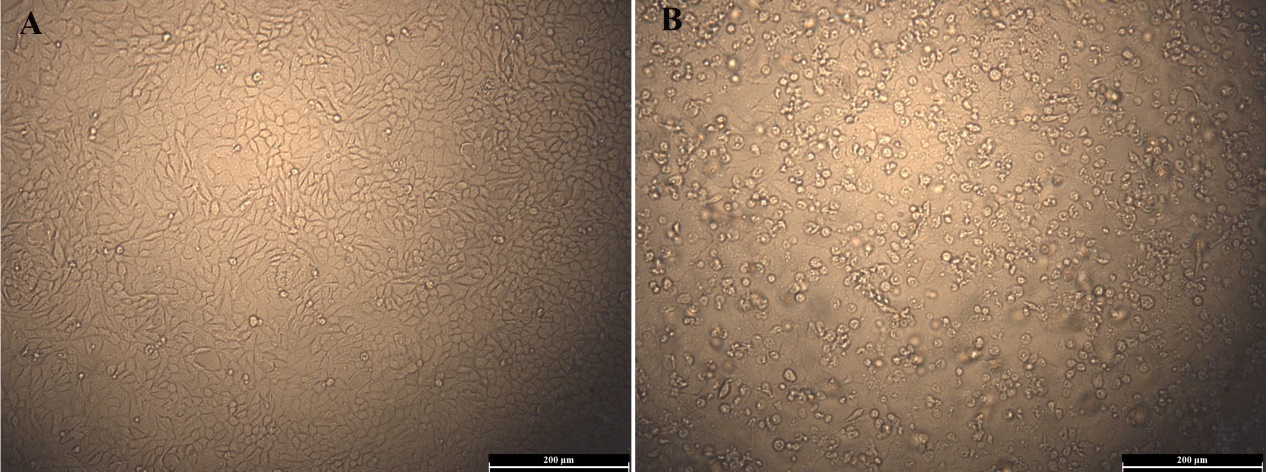


Fig S2 Cytopathic effect induced by SVA infection

A, Control BHK-21 cells; B, BHK-21 cells incubated with samples

| Table S1 Rectal temperature detected daily | | | | | | |
| --- | --- | --- | --- | --- | --- | --- |
| dpi | Pig 1 (℃) | Pig 2 (℃) | Pig 3 (℃) | Control 1 (℃) | Control 2 (℃) | Control 3 (℃) |
| -1 | 38.8 | 39.0 | 39.0 | 38.8 | 38.6 | 38.8 |
| 0 | 39.5 | 39.4 | 39.7 | 39.3 | 39.2 | 39.1 |
| 1 | 39.8 | 39.7 | 39.6 | 39.2 | 39.5 | 39.3 |
| 2 | 39.7 | 39.8 | 39.4 | 39.5 | 39.4 | 39.4 |
| 3 | 39.2 | 39.3 | 39.4 | 39.9 | 39.7 | 39.7 |
| 4 | 39.6 | 40.3 | 39.3 | 39.7 | 39.3 | 39.4 |
| 5 | 39.8 | 39.6 | 39.5 | 39.9 | 39.9 | 40.3 |
| 6 | 39.7 | 39.3 | 39.1 | 39.7 | 39.5 | 39.6 |
| 7 | 39.9 | 38.5 | 38.2 | 39.6 | 39.4 | 40.1 |
| 8 | 39.2 | 39.2 | 39.3 | 38.9 | 39.5 | 39.2 |
| 9 | 39.6 | 39.3 | 39.4 | 38.8 | 39.4 | 39.6 |
| 10 | 39.2 | 39.5 | 39.0 | 38.2 | 38.3 | 39.3 |
| 11 | 38.8 | 39.1 | 38.0 | 38.6 | 39.0 | 38.2 |
| 12 | 38.6 | 39.3 | 39.0 | 38.7 | 39.0 | 38.6 |
| 13 | 38.7 | 39.0 | 39.1 | 38.9 | 39.2 | 38.8 |
| 14 | 38.6 | 39.1 | 38.9 | 38.6 | 38.6 | 38.6 |

| Table S2 SVA genomic RNA quantification in serum by TaqMan-based qRT-PCR | |
| --- | --- |
| dpi^a^ | SVA RNA quantification /lg (genomic copies per μL) |
| 1 | 3.921±0.159 |
| 3 | 4.812±0.889 |
| 5 | / |
| 7 | / |
| 10 | / |
| 14 | / |
| a: Days post infection;  b: SVA RNA quantification, {lg (genomic copies per μL), mean±SD};  / : Not detected. | |

Table S3 SVA antibody detected by cELISA method

| dpi | OD | Percentage of inhibition (%) | OD | Percentage of inhibition (%) | OD | Percentage of inhibition (%) | Control | OD |
| --- | --- | --- | --- | --- | --- | --- | --- | --- |
| 1 | 1.684069 | 6.18 | 3.022904 | 1.34 | 5.426112 | 31.42 | positive | 0.088 |
| 3 | 1.447129 | 19.38 | 2.597597 | 49.46 | 4.662686 | 40.28 | positive | 0.109 |
| 5 | 0.338717 | 81.13 | 0.607996 | 75.97 | 1.091353 | 91.46 | negative | 1.838 |
| 7 | 0.152216 | 91.52 | 0.273228 | 83.75 | 0.490444 | 93.05 | negative | 1.751 |
| 10 | 0.117393 | 93.46 | 0.21072 | 92.67 | 0.378243 | 92.48 | / | / |
| 14 | 0.134984 | 92.48 | 0.242296 | 90.82 | 0.434922 | 92.03 | / | / |

| Table S4 Neutralizing antibody titer of pigs inoculated with SVA CH/FuJ/2017 via intramuscular after infection | | | |
| --- | --- | --- | --- |
| dpi^a^ | Pig number | | |
|  | No. 1 | No.2 | No.3 |
| 1 | / | / | / |
| 3 | / | / | / |
| 5 | / | / | / |
| 7 | 1:80 | 1:46.90 | 1:35.49 |
| 10 | 1:254.13 | 1:80 | 1:187.65 |
| 14 | 1:320 | 1:95 | 1:225.99 |
| a: Days post infection;  -/: Not detected. | | | |
